# Transanal Intubation for Preventing Colorectal Anastomotic Failure

**DOI:** 10.1155/2024/5562420

**Published:** 2024-08-10

**Authors:** Mykola Gordiichuk

**Affiliations:** ^1^ Shupyk National University of Healthcare of Ukraine, Kyiv, Ukraine; ^2^ Communal Nonprofit Enterprise Kyiv City Clinical Oncology Center, Kyiv, Ukraine

## Abstract

**Introduction:**

Failure of low colorectal anastomosis remains challenging in surgical oncology, necessitating the exploration of new methods and improvements in existing preventive measures.

**Materials and Methods:**

This prospective study was conducted in two stages: intraluminal pressure in the colon was monitored in 32 patients by manometry and sonography over a 5-day postoperative period; 213 patients who underwent anterior resection of the rectum were analyzed, of whom 126 and 87 underwent diverting stoma (DS) and transanal intubation (TAI), respectively.

**Results:**

The effectiveness of the recommended technique for applying and removing transanal intubation (TAI) to prevent pneumo hydro strike (≥15 kPa) on the anastomosis line was analyzed in 87 patients and compared with imposed DS. TAI showed better borderline statistical significance (*p* = 0.051). The incidence of repeat surgery for anastomotic failure (AL) was seven (5.55%) and four (4.59%) in the DS and TAI groups, respectively. The distance of the anastomosis from the dentate line <60 mm was associated with a higher risk of AL occurrence (odds ratio (OR), 1.012; 95% confidence interval (CI), 1.007–1.017; *p* < 0.001; area under the curve (AUC) = 0.82). DS is recommended for men, as the risk of AL is significantly lower among women (OR, 0.41; 95% CI, 0.16–1.04; *p* = 0.062; AUC, 0.61; 95% CI, 0.54–0.67).

**Conclusions:**

Although TAI is advantageous over DS for preventing AL, surgeons select the method for the preventive approach based on the preoperative and intraoperative results.

## 1. Introduction

Patients with rectal cancer exhibit high morbidity and mortality rates, often requiring appropriate treatment. According to the WHO International Agency for Research on Cancer, colorectal cancer ranks third globally in terms of cancer incidence and second in terms of mortality (9.4%) [1, 2]. The incidence rate of rectal cancer in Ukraine has been increasing from 16.8 to 19.4 cases per 100,000 individuals, and the mortality rate has increased from 10.3 to 11.6 per 100,000 individuals [3].

Surgical treatment, considered the second stage of complex treatment, remains the “gold standard” for rectal cancer. This enables radical removal of the primary tumor with potential routes of metastasis and provides valuable diagnostic information regarding the effectiveness of the neoadjuvant therapy, including the degree of tumor regression, number of involved lymph nodes, presence or absence of lymphovascular and perineural invasion, and extranodal tumor deposits.

Despite the current developments in oncological science and surgical strategies, the risk of anastomotic failure (AL) remains high. AL is one of the severe complications that occurs after the formation of a colorectal anastomosis and has been reported in 3.5–21% of patients, with an associated postoperative mortality rate of 6.0–39.3% [4–6]. AL results in peritonitis, sepsis, increased mortality, prolonged hospitalization, high rate of recurrence, risk of permanent stoma, increased financial burden on the healthcare system, and poor quality of life [7–10]. Commonly accepted methods for preventing the failure of colorectal anastomosis include the formation of a diverting stoma (DS) or ileostomy [11, 12], which do not fully resolve this issue. Preventive stoma does not reduce the risk of failure of the colorectal anastomosis but only contributes to the prevention of severe complications, thereby reducing the need for repeated operations and relaparotomy [8, 9, 13, 14]. An alternative method of applying DS is the use of a transanal tube, which facilitates drainage on the proximal side of the anastomosis and reduces the risk of intestinal content extrusion onto the line of the formed anastomosis, thereby reducing the frequency of AL. The presented technique of placing the tube differs in nonessential details, specifically in the diameter of the tube and the distance at which the proximal end of the tube was placed (30–70 mm above the formed anastomosis), which is usually removed on postoperative days 5–7 [15–18]. The recommended positioning of the proximal edge of the tube and the duration of its placement have several limitations that require further study and refinement.

### 1.1. Aim

This study's aim was to evaluate the outcomes of colorectal AL using transanal intubation (TAI) with recommended placement and removal techniques.

## 2. Materials and Methods

This prospective study was conducted in two stages. In the first stage, during 2019, the primary objective was to determine the optimal technique for placing, maintaining, and removing a TAI in patients with rectal cancer with colorectal anastomosis (approved by the Ethics Commission of the National University of Health Care of Ukraine (protocol No. 3 dated February 5, 2019)). In 32 patients, within 5 days of the early postoperative period, phasic and tonic contractile activity and movement of intraluminal contents in the colon were assessed using intraoperative retrograde placement of solid-state manometric catheters. These catheters, equipped with 16 strain gauges at an interval of 7.5 cm, were inserted with the tip positioned in the cecum. The recording from this catheter was captured using a portable recording device known as a datalogger (RedTech, Inc., Calabasas, CA, USA), with a sampling rate of 8 Hz and a 32 MB flash card memory capacity. Concurrent sonographic monitoring (Ultrasonograf, Toshiba Aplio MX) was performed during the study period. The results revealed that intra-intestinal pressure is formed in the colon in the period from 58.9 ± 3.1 to 73.7 ± 6.7 h postoperatively, which leads to the occurrence of pneumo hydro strike (≥15 kPa) on the anastomosis line.

The second stage of this study was conducted between 2020 and 2023. The objective was to evaluate the effect of the proposed placement and removal of TAI on the frequency and nature of colorectal AL. The study included 216 patients with rectal cancer who underwent anterior rectal resection during the second stage of complex treatment, among whom 129 and 87 underwent DS and TAI, respectively. The inclusion criteria were as follows: age ≥18 years; morphologically confirmed adenocarcinoma of various grades of differentiation; localization of the tumor within ≤100 mm from the anorectal angle based on magnetic resonance imaging; stages II-III (T3-4N0M0, TanyN1-2M0); patients who had completed a course of neoadjuvant chemoradiotherapy (CRT) 44–55 Gy and two cycles of capecitabine or 5-fluorouracil infusion; and those who had undergone an anterior or low anterior rectal resection within 7 to 10 weeks after completion of radiation therapy. Patients who did not use methods to prevent AL, those with no anastomosis after surgery, and those with postoperative complications that were not related to AL were excluded.

Three patients with DS who refused to participate were excluded from the study.

The identified failure of colorectal anastomosis was characterized according to the classification of the International Study Group of Rectal Cancer [19] (Table 1).

Patients in whom AL was not diagnosed on the seventh postoperative day, contrast examination using radiography of the formed anastomosis site was performed using the Apollo EZ device (2018, Italy). The contrast agent (100 mL of 76% solution with an iodine concentration of 370 mg/mL) was injected trans-anally using a silicone Foley catheter No. 14 (with the cuff inflated to 5 mL). Four radiographs were obtained in the frontal and lateral projections with tight filling and emptying, and leakage of the contrast outside the intestinal lumen was ascertained as a grade A failure.

The data were entered into an Excel spreadsheet. All statistical analyses were performed using EZR v. 1.54 (graphical user interface for R statistical software version 4.0.3, R Foundation for Statistical Computing, Vienna, Austria) [20]. Quantitative results were presented as mean ± standard deviation (for normally distributed data) or the median value and interquartile range (for a skewed distribution), and Student's *t*-test (for a normal distribution) or Mann–Whitney *U* test was used for comparison (for a skewed distribution). Chi-square and Fisher's exact tests were used to compare the distribution of qualitative variables. Logistic regression models were constructed to predict the risk of early treatment failure. The risk of progression is presented as odds ratios (OR) with corresponding 95% confidence intervals (CI). Factors with *p* < 0.05 in the analysis were considered statistically significant.

This clinical study was approved by the Ethics Commission of the National University of Health Care of Ukraine (protocol No. 14 dated 07.12.2020).

## 3. Results

**Figure 1:**
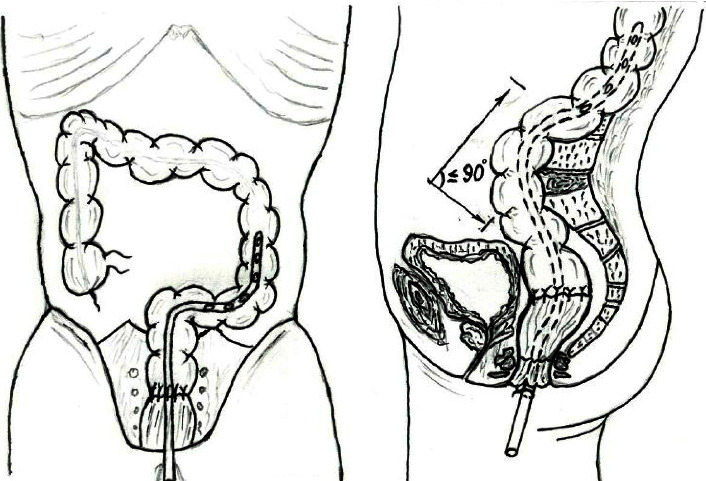
Placement of the transanal intubation tube: (a) frontal; (b) lateral [21].

Based on the results of the first stage of the study and considering the features of the anastomosis placement, specifically the anatomical landmarks such as the promontorium and lordosis, with physiological placement of the plane of the formed anastomosis to the lumen of the adduction loop at an angle of ≤90°, the proximal end of the TAI tube was positioned in the adduction loop above the anastomosis to prevent the direct impact of pneumo hydro strike on the formed anastomosis. The tube F: 24–30 was used, with its proximal end positioned at 25–45 cm (33.4 ± 4.7) above the anastomosis, with a nonspringy, free placement within the proximal loop of the intestine, and the distal end was secured to the perineum (Figure 1).

From postoperative day 2 onward, gases were released through the tube, followed by liquid masses. The TAI was removed at 88.2 ± 6.3 hours postoperatively. To facilitate this, the tube was gradually introduced into the lumen for 7–10 min, during which 40 mL of petroleum jelly and 100 mL of metronidazole were administered. After 10–15 min, defecation with gases, chyme, and liquid feces began, which lasted up to 2 h, with an average volume of 1.4 ± 0.23 kg, followed by regular bowel movements.

During the second stage, based on the inclusion, noninclusion, and exclusion criteria, the patients were divided into two groups: the main group, which included 87 patients who had undergone TAI to prevent the failure of a low colorectal anastomosis, and the comparison group comprising 126 patients who had undergone DS (Table 2).

The clinical characteristics of the groups according to sex, average age, median body weight, stage of the process, grade of tumor differentiation, method, and level of anastomosis formation were randomized and no statistically significant differences were found between the groups.

Clinical, radiographic, sonographic, endoscopic, and laboratory examinations of 213 patients after the formation of a low colorectal anastomosis in the early postoperative period revealed AL in 22 patients (10.33%). In the group with imposed DS, AL was reported in 18 patients (14.28%) with severity grades A, B, and C in 7 (5.55%), 4 (3.17%), and 7 (5.55%), respectively. In the group with TAI, 4 patients (4.59%) were diagnosed with AL (grade C severity in all patients, with no grade A or B). Failure in both groups was diagnosed between the fifth and seventh postoperative days, with the exception of one case in a patient with TAI, which was diagnosed on the 10^th^ day and manifested as a rectovaginal fistula.

During the outpatient follow-up, AL was endoscopically diagnosed in three patients, 54.6 ± 7.4 days after surgical treatment, when they were examined prior to closing the stoma, which was characterized by a defect on the line of anastomosis with an inflammatory sinus, which prevented restoration of intestinal continuity. Studies conducted during the postoperative period revealed no deviations in patients without AL. Patients with late AL were assigned to group D. Therefore, in the DS group, AL was categorized into grades A, B, C, and D in 7, 4, 11, and 3 patients, respectively. The general distribution of AL is presented in Table 3.

Our results indicated the superior efficacy of TAI over DS in preventing AL (*p* = 0.051). The need for emergency medical measures in patients with AL was as follows: patients in groups A and D received no intervention; 4 patients in group B received adjusted drug therapy (detoxification and antibiotic therapy) and interventional procedures; and 11 patients in group C underwent repeated surgery. Therefore, repeat surgery for AL was required in seven (5.55%) and four patients (4.59%) in the DS and TAI groups, respectively, with no significant differences between the groups.

Logistic regression models were constructed to predict AL risk.  Table 4 shows the results of the univariate analysis of failure in 25 patients (cases, *Y* = 1), compared with 188 patients without failure (noncases, *Y* = 0).

These findings indicate that the frequency of AL occurrence was significantly lower in patients who had undergone TAI than in those who had undergone DS (*p*=0.051), and women had a significantly lower risk of AL (*p*=0.062). Age (*p*=0.189) and body mass index (BMI) (*p*=0.693) were not significantly associated with AL. Compared with an anastomosis formed at a level ≥60 mm, AL risk was higher in the anastomosis formed at a distance <60 mm to the dentate line (OR, 1.012; 95% CI, 1.007–1.017; *p* < 0.001; area under the curve (AUC) = 0.82).

## 4. Discussion

The violation of the issues of prevention, early diagnosis, and selection of treatment strategy for AL in patients with distal rectal cancer is actively discussed and will remain relevant for the foreseeable future. Despite the availability of various approaches to reduce AL incidence, no substantial breakthroughs have been reported. A technique for the placement and removal of TAI with justification for the length of stay is proposed in this study. The results were compared with those obtained using the traditional method of preventing AL with imposed DS. The conventional method has several disadvantages such as the increase in the duration of surgical intervention, risk of paracolostomy dermatitis or hernia, intestinal obstruction, and intussusception of the intestine due to DS, and its closure requires a repeat operation with anesthesia, which can lead to complications [22, 23]. A recommended alternative is a transanal tube to decrease the intraluminal pressure on the proximal side and an anastomosis line to reduce quantitative failure [24–27]. The study findings demonstrated that pneumo hydro strike (≥15 kPa) is formed in the lumen of the colon between the second and third postoperative days; therefore, to prevent its effect on the anastomosis line, TAI was performed with decompression of the adductor loop of the intestine before the anastomosis. The TAI tube was removed 88.2 ± 6.3 hours after surgery using a technique that ensured further regular bowel movements. The proposed technique reduced the TAI stay by almost half of the recommended duration [15–18], yielding better outcomes in terms of AL incidence [28, 29], especially among patients who had received neoadjuvant CRT.

The incidence of AL was significantly lower in the TAI group than in the DS group (5 (5.75%) vs. 21 (16.66%); OR, 0.36; 95% CI, 0.13–1.00; *p*=0.051; AUC, 0.61; 95% CI, 0.54–0.68). However, the grade of severity classified as “C” that required repeated operations was comparable between the groups (7 (5.55%) with DS and 4 (4.59%) with TAI). Late AL failure categorized as grade D was reported in three (2.38%) patients in the DS group at 54.6 ± 7.4 days during the outpatient examination; however, when examined according to the study protocol, they were initially assigned to the group without AL. Expanding the cohort of patients who underwent surgery for rectal cancer to the DS group may significantly increase the frequency of grade D AL. Currently, the timing of their occurrence remains unclear; however, it cannot be excluded because they can be attributed to AL grade A, which is not typically diagnosed on the seventh day. Further studies are needed to understand the underlying causes of their occurrence, including post-radiation changes in the intestine, rheological and functional changes in the disconnected segment of the intestine, and microbial factors affecting the protective qualities of the mucous membrane.

Grade C AL often requires emergency surgeries. For AL grades A, B, and D, endoscopic, minimally invasive manipulations were performed, which required a long time [30, 31] to close the DS and restore the patient's quality of life.

Therefore, the choice of AL prevention method by the surgeon was determined based on preoperative and intraoperative examinations. DS can be recommended in men, as the risk of its occurrence is lower among women (OR, 0.41; 95% CI, 0.16–1.04; *p*=0.062; AUC, 0.61; 95% CI, 0.54–0.67). Additionally, the risk of anastomosis is significantly higher when the anastomosis line is < 60 mm from the dentate line (OR, 1.012; 95% CI, 1.007–1.017; *p* < 0.001; AUC, 0.82; 95% CI, 0.76–0.87). These findings emphasize that the technique selection was not influenced by patient age (*p*=0.189) or BMI (*p*=0.693).

## 5. Conclusions

This study elucidated the effectiveness of TAI at low colorectal anastomosis, which mitigated the effect of pneumo-hydrostrike on the anastomosis line by reducing the intra-intestinal pressure in the proximal bowel loop, thereby decreasing the rate of AL. This method can be recommended to prevent AL during surgery in patients with rectal cancer.

## Figures and Tables

**Table 1:** Grading of colorectal anastomosis failure according to the classification of the International Study Group of Rectal Cancer.

Grade of severity	Clinical presentation
А-radiological	The condition is detectable only by radiation methods, there are no clinical and laboratory symptoms, it does not require additional treatment
В-clinical symptoms	Has clinical symptoms with laboratory changes, requires active medical treatment (antibiotics, detoxification therapy), surgical manipulations
С-clinically featured (peritonitis, sepsis)	Severe clinical symptoms with laboratory changes, peritonitis is diagnosed, requiring relaparotomy

**Table 2:** Patient characteristics depending on the method of prevention of anastomosis failure.

Parameter	Diverting stoma (*n* = 126)	Transanal intubation (*n* = 87)	*p*
Gender, *n* (%):			
Female	64 (50.8)	41 (47.1)	0.781
Male	62 (49.2)	46 (52.9)	
Mean age ± *σ* (min-max), years	66.9 ± 8.4	67.1 ± 9.3	0.283
Median body mass index (min-max), kg/m^2^	26.7 (24.3–29.0)	27.4 (24.2–29.5)	0.526
pTNM stage, *n* (%):			
II (рТ3–4N0М0)	92 (73.02)	63 (72.41)	0.792
III (рТ2–4N1–2М0)	34 (26.98)	24 (27.59)	
Tumor differentiation grade, *n* (%):			
G1, G2	109 (86.51)	76 (87.36)	0.801
G3 + mucosal adenocarcinoma	17 (13.49)	11 (12.64)	
Method of anastomosis formation, *n* (%):			
Stapler	97 (76.98)	61 (70.11)	0.298
Manual	29 (23.02)	26 (29.89)	
The level of the formed anastomosis to the dentate line (mm), *n* (%):			
<60	101 (80.16)	66 (75.82)	0.308
≥60	25 (19.84)	21 (24.14)	

Student's *t*-test (in the case of a normal distribution) or Mann–Whitney *U* test (in the case of a nonnormal distribution) was used for comparison. Fisher's exact test was used to compare the distributions of qualitative features.

**Table 3:** Groupwise distribution of the severity of the anastomosis failure.

Grade of severity	Diverting stoma (*n* = 126)	Transanal intubation (*n* = 87)	*p*
А, *n* (%)	7 (5.55)	0 (0)	=0.051
В, *n* (%)	4 (3.17)	0 (0)	
С, *n* (%)	7 (5.55)	4 (4.59)	
D (late), *n* (%)	3 (2.38)	0 (0)	
Together, *n* (%)	21 (16.66)	4 (4.59)	

The chi-square test was used to compare the distribution of qualitative features.

**Table 4:** One-factor logistic regression models for predicting the risk of failure.

Risk factor		Odds ratio, OR (95% СІ)	The level of significance of the difference OR from 1, *p*	Area under the operating characteristics curve, AUC (95% СІ)
Group	DS	Reference		0.61 (0.54–0.68)
	TAI	0.36 (0.13–1.00)	0.051	

Sex	M	Reference		0.61 (0.54–0.67)
	F	0.41 (0.16–1.04)	0.062	

Age		—	0.189	0.59 (0.52–0.66)

BMI		—	0.693	—

The level of the anastomosis to the dentate line (mm)	≥60	Reference		
	<60	1.012 (1.007–1.017)	<0.001	0.82 (0.76–0.87)

OR >1 indicates increased risk and OR <1 indicates decreased risk.

## Data Availability

Data supporting the findings of this study are included in this article. However, other data are not freely available; justifications for restricting access may include legal and ethical concerns, such as patient privacy and commercial confidentiality.

## References

[1] Bray F., Ferlay J., Soerjomataram I., Siegel R. L., Torre L. A., Jemal A. (2018). Global cancer statistics 2018: GLOBOCAN estimates of incidence and mortality worldwide for 36 cancers in 185 countries. *CA: A Cancer Journal for Clinicians*.

[2] Sung H., Ferlay J., Siegel R. L. (2021). Global cancer statistics 2020: GLOBOCAN estimates of incidence and mortality worldwide for 36 cancers in 185 countries. *CA: A Cancer Journal for Clinicians*.

[3] Fedorenko Z. P., Gulak L. . О., Mykhailovych Y. I. (2021). Cancer in Ukraine, 2019–2020, Morbidity, mortality, performance indicators of the oncology service. *Bulletin of the National Chancery Register of Ukraine*.

[4] Jestin P., Pahlman L., Gunnarsson U. (2008). Risk factors for anastomotic leakage after rectal cancer surgery: a case–control study. *Colorectal Disease*.

[5] Law W. l, Chu K. W., Ho J. W., Chan C. W. (2000). Risk factors for anastomotic leakage after low anterior resection with total mesorectal excision. *The American Journal of Surgery*.

[6] Peeters K. C., Tollenaar R. A., Marijnen C. A. (2005). Risk factors for anastomotic failure after total mesorectal excision of rectal cancer. *British Journal of Surgery*.

[7] Nesbakken A., Nygaard K., Lunde O. C. (2002). Outcome and late functional results after anastomotic leakage following mesorectal excision for rectal cancer. *British Journal of Surgery*.

[8] Boccola M. A., Buettner P. G., Rozen W. M. (2011). Risk factors and outcomes for anastomotic leakage in colorectal surgery: a single-institution analysis of 1576 patients. *World Journal of Surgery*.

[9] Kang C. Y., Halabi W. J., Chaudhry O. O. (2013). Risk factors for anastomotic leakage after anterior resection for rectal cancer. *JAMA Surgery*.

[10] Law W. L., Choi H. K., Lee Y. M., Ho J. W., Seto C. L. (2007). Anastomotic leakage is associated with poor long-term outcome in patients after curative colorectal resection for malignancy. *Journal of Gastrointestinal Surgery*.

[11] Tan W. S., Tang C. L., Shi L., Eu K. W. (2009). Meta-analysis of defunctioning stomas in low anterior resection for rectal cancer. *British Journal of Surgery*.

[12] Hüser N., Michalski C. W., Erkan M. (2008). Systematic review and meta-analysis of the role of defunctioning stoma in low rectal cancer surgery. *Annals of Surgery*.

[13] Shogan B. D., Carlisle E. M., Alverdy J. C., Umanskiy K. (2013). Umanskiy K Do we really know why colorectal anastomoses leak?. *Journal of Gastrointestinal Surgery*.

[14] Wong N. Y., Eu K. W. (2005). A defunctioning ileostomy does not prevent clinical anastomotic leak after a low anterior resection: a prospective, comparative study. *Diseases of the Colon & Rectum*.

[15] Xiao L., Zhang W. B., Jiang P. C. (2011). Can transanal tube placement after anterior resection for rectal carcinoma reduce anastomotic leakage rate? A single-institution prospective randomized study. *World Journal of Surgery*.

[16] Kim M. K., Won D. Y., Lee J. K., Kang W. K., Kim J. G., Oh S. T. (2015). Comparative study between transanal tube and loop ileostomy in low anterior resection for mid rectal cancer: a retrospective single center trial. *Annals Surgery Treatment Research*.

[17] Zhao W. T., Hu F. L., Li Y. Y., Li H. J., Luo W. M., Sun F. (2013). Use of a transanal drainage tube for prevention of anastomotic leakage and bleeding after anterior resection for rectal cancer. *World Journal of Surgery*.

[18] Nishigori H., Ito M., Nishizawa Y. (2014). Effectiveness of a transanal tube for the prevention of anastomotic leakage after rectal cancer surgery. *World Journal of Surgery*.

[19] Rahbari N. N., Weitz J., Hohenberger W. (2010). Definition and grading of anastomotic leakage following anterior resection of the rectum: a proposal by the International Study Group of Rectal Cancer. *Surgery*.

[20] Kanda Y. (2013). Investigation of the freely available easy-to-use software “EZR” for medical statistics. *Bone Marrow Transplantation*.

[21] Gordiichuk M. P. Method of transanal intubation to prevent failure of colorectal anastomosis in patients operated on for rectal cancer/A61B17/11, A61M25; 2022-08-03: byul.N 31. https://sis.ukrpatent.org/uk/search/detail/1700643.

[22] Cho S. H., Lee I. K., Lee Y. S., Kim M. K. (2021). The usefulness of transanal tube for reducing anastomotic leak in mid rectal cancer: compared to diverting stoma. *Annals Surgery Treatment Research*.

[23] Floodeen H., Hallböök O., Hagberg L. A., Matthiessen P. (2017). Costs and resource use following defunctioning stoma in low anterior resection for cancer: a long-term analysis of a randomized multicenter trial. *European Journal of Surgical Oncology*.

[24] Zhao W. T., Li N. N., He D., Feng J. Y. (2017). Transanal tube for the prevention of anastomotic leakage after rectal cancer surgery: a systematic review and meta-analysis. *World Journal of Surgery*.

[25] Goto S., Hida K., Kawada K. (2017). Multicenter analysis of transanal tube placement for prevention of anastomotic leak after low anterior resection. *Journal of Surgical Oncology*.

[26] Wang F. G., Yan W. M., Yan M., Song M. M. (2019). Comparison of anastomotic leakage rate and reoperation rate between transanal tube placement and defunctioning stoma after anterior resection: a network meta-analysis of clinical data. *European Journal of Surgical Oncology*.

[27] Ito T., Obama K., Sato T. (2017). Usefulness of transanal tube placement for prevention of anastomotic leakage following laparoscopic low anterior resection: transanal tube prevents AL following LLAR. Asian J. Endosc. *Asian Journal of Endoscopic Surgery*.

[28] Strangio G., Zullo A., Ferrara E. C. (2015). Endo-sponge therapy for management of anastomotic leakages after colorectal surgery: a case series and review of literature. *Digestive and Liver Disease*.

[29] Verlaan T., Bartels S. A., van Berge Henegouwen M. I., Tanis P. J., Fockens P., Bemelman W. A. (2011). Early, minimally invasive closure of anasto-motic leaks: a new concept. *Colorectal Disease*.

[30] Nerup N., Johansen J. L., Alkhefagie G. A., Maina P., Jensen K. H. (2013). Promising results after endoscopic vacuum treatment of anastomotic leakage following resection of rectal cancer with ileostomy. *Danish Medical Journal*.

[31] Chen Y. S., Bo X. B., Gu D. Y., Gao W. D., Sheng W. Z., Zhang B. (2015). Outcomes of laparoscopic abdominoperineal resection in low rectal cancer using different pelvic drainages. *Asian Pacific Journal of Cancer Prevention*.

